# Case Report: A New Family With Pontocerebellar Hypoplasia 10 From Sudan

**DOI:** 10.3389/fgene.2022.883211

**Published:** 2022-06-02

**Authors:** Mutaz Amin, Cedric Vignal, Ahlam A. A. Hamed, Inaam N. Mohammed, Maha A. Elseed, Rayan Abubaker, Yousuf Bakhit, Arwa Babai, Eman Elbadi, Esraa Eltaraifee, Doua Mustafa, Ashraf Yahia, Melka Osman, Mahmoud Koko, Mohamed Mustafa, Mohamed Alsiddig, Sahwah Haroun, Azza Elshafea, Severine Drunat, Liena E. O. Elsayed, Ammar E. Ahmed, Odile Boespflug-Tanguy, Imen Dorboz

**Affiliations:** ^1^ Faculty of Medicine, Al-Neelain University, Khartoum, Sudan; ^2^ INSERM UMR 1141 PROTECT, Université Paris Diderot-Sorbonne, Paris, France; ^3^ Unité de Génétique Moleculaire, Departement de Genetique Médicale, APHP, Hopital Robert-Debré, Paris, France; ^4^ Faculty of Medicine, University of Khartoum, Khartoum, Sudan; ^5^ Neurogenetics Research group, Faculty of Medicine, University of Khartoum, Khartoum, Sudan; ^6^ National University Biomedical Research Institute, National University-Sudan, Khartoum, Sudan; ^7^ Faculty of Dentistry, University of Khartoum, Khartoum, Sudan; ^8^ Department of Neurobiology, Centre for Neurology, UKB, University of Bonn, Bonn, Germany; ^9^ Department of Basic Sciences, College of Medicine, Princess Nourah bint Abdulrahman University, Riyadh, Saudi Arabia; ^10^ Neuropediatrics and Metabolic Disorders Department, Reference Center for Leukodystrophies and Rare Leukoencéphalopathies (LEUKOFRANCE), CHU APHP Robert-Debré, Paris, France

**Keywords:** pontocerebellar hypoplasia 10, CLP1, Sudan, pontocerebellar hypoplasia, family

## Abstract

Pontocerebellar hypoplasia type 10 (PCH10) is a very rare autosomal recessive neurodegenerative disease characterized by intellectual disability, microcephaly, severe developmental delay, pyramidal signs, mild cerebellar atrophy, and white matter changes in the brain, as shown by magnetic resonance imaging (MRI). The disease has been described in only twenty-one patients from ten Turkish families with a founder missense pathogenic variant in the *CLP1* gene involved in tRNA processing and maturation. We analyzed three siblings from a consanguineous Sudanese family who presented with intellectual disability, dysmorphic features, developmental delay, regression of milestones, microcephaly, epilepsy, extrapyramidal signs, mild pontine, and cerebellar atrophy. We identified through whole-exome sequencing the same pathogenic variant (c.419G>A; p(Arg140His) reported before in all Turkish families. Our study extends the phenotypes of PCH10 and reports for the first time cases with PCH10 of non-Turkish origin.

## Introduction

Pontocerebellar hypoplasia (PCH) describes a heterogeneous group of rare neurodegenerative disorders characterized by developmental delay, epilepsy, spasticity, and postnatal microcephaly (Rudnik-Schöneborn et al., 2014). Some patients have dysmorphic facial features and axonal sensorimotor neuropathy (Van Dijk et al., 2017; Morton et al., 2018; Somashekar et al., 2021). Brain MRI of patients with PCH reveals the characteristic atrophy of the pons and cerebellum. In most cases, death is invariable within the first decade of life (Van Dijk et al., 2018).

There are currently 13 different types of pontocerebellar hypoplasia resulting from mutations in at least 19 different genes (Rüsch et al., 2020). Many of these genes are involved in RNA processing which is essential for neuronal survival (Van Dijk et al., 2018). PCH1 and PCH2 are the most common types; they are caused by mutations in *EXOSC3*, *TSEN54*, *RARS2*, and *VRK1* genes (Van Dijk et al., 2018).

Pontocerebellar hypoplasia type 10 (PCH10) is a very rare and recently described type of PCH. It presents with microcephaly, severe developmental delay, pyramidal signs, and very mild cerebellar atrophy and white matter changes in brain MRI (Karaca et al., 2014; Schaffer et al., 2014; Wafik et al., 2018). The disease is caused by a pathogenic variant in the *CLP1* gene, which encodes a kinase involved in tRNA processing and maturation (Karaca et al., 2014; Weitzer et al., 2015). Dysfunction of the CLP1 enzyme leads to the accumulation of aberrant tRNA molecules and ultimately to neuronal death (Morisaki et al., 2021).

Only ten families have been reported so far with PCH10 (Karaca et al., 2014; Schaffer et al., 2014; Wafik et al., 2018). All families originated from Turkey, and all shared the same mutation (c.419G>A; p.Arg140His) (Karaca et al., 2014; Schaffer et al., 2014; Wafik et al., 2018). The pathogenicity of the R140H variant has been functionally validated in several studies with the resulting defect in kinase activity (Karaca et al., 2014; Schaffer et al., 2014; Weitzer et al., 2015; Hayne et al., 2020; Monaghan et al., 2021; Morisaki et al., 2021). The associated neurodegenerative phenotype has also been reproduced in both mice (Monaghan et al., 2021; Morisaki et al., 2021) and Zebrafish models (Schaffer et al., 2014).

This study reports for the first time a non-Turkish family with PCH10 from Sudan sharing the same pathogenic variant.

### Case Presentation

**FIGURE 1:**
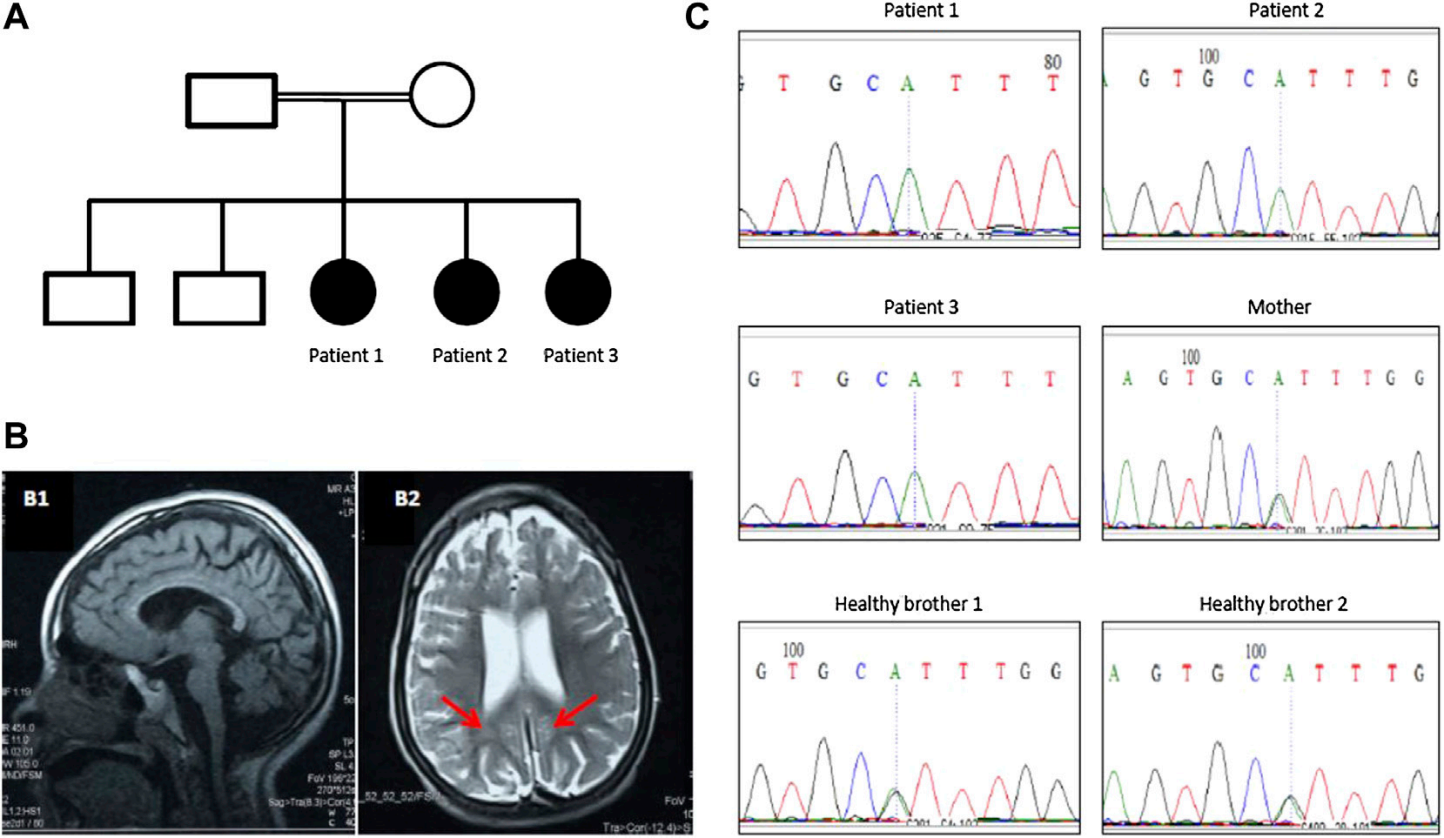
**(A)** Family pedigree of a Sudanese family with PCH10. **(B)** Brain MRI of patient 1 showing mild pontine and cerebellar atrophy and thin corpus callosum (B1) and periventricular white matter changes (red arrows in B2). **(C)** Segregation of CLP1 (c.419G>A; p.Arg140His) pathogenic variant in the family with pontocerebellar hypoplasia 10.

Three sisters born to first-degree consanguineous parents were studied (Table 1). The first (patient 1) was 8 years old and presented with delayed motor and speech development (sitting at 18 months, crawling at 1 year, disyllabic babbling only). She started to regress at 5 years (lost walking and disyllabic babbling). She had behavioral abnormality (irritability, biting), mild cognitive impairment, and epilepsy since the age of 11 months, controlled with antiepileptic medications. There was no sphincteric control. Patient 2 (7 years) had a severely delayed motor and speech development (sitting at 21 months, no standing or walking, no speech) but no regression. Patient 3 (1 year) had a delayed motor and speech development (head support at 7 months, sitting without support at 8 months, no standing or walking, and no speech). On examination, all patients had mild dysmorphic features, including high arched eyebrows, long palpebral fissures and eyelashes with prominent eyes, microcephaly (<3SD), hypertonia, hyperreflexia, dystonia, extrapyramidal signs (uncontrollable head nodding), poor dentition, and fixed flexion deformity (Table 1). Brain MRI showed mild pontine and cerebellar atrophy, thin corpus callosum, and periventricular white matter changes (Figure 1). The metabolic screening was negative.

**TABLE 1:** Clinical features of patients with *CLP1* mutation.

	Reported patients (Turkey)	This study (Sudan)		
No.	21	Patient 1	Patient 2	Patient 3
Age of diagnosis (mean)	3.9	8	7	1
Gender (M/F)	5/6	F	F	F
Intellectual disability	20/20	+	+	+
Microcephaly	20/20	+	+	+
Dysmorphism	10/13	+	+	+
Delayed motor and speech development	21/21	+	+	+
Developmental regression	0/21	+	-	NA
Epilepsy	17/21	+	-	-
Spasticity	15/20	+	+	+
Peripheral neuropathy	8/12	NA	NA	NA
Behavioral abnormalities	Not reported	+	-	NA
MRI findings				
Cortical atrophy	15/19	+	+	NA
Cerebellar atrophy	11/19	+	+	NA
Pontine atrophy	6/19	+	+	NA
White matter changes	8/19	+	+	NA
Thin corpus callosum	13/19	+	+	NA

### Genetic Testing

#### DNA Extraction

Two ml of saliva was collected from patients and other healthy family members using DNA Oragene Saliva kits (DNA Genotek Inc., Ottawa, ON, Canada). DNA extraction was done according to the prepIT.L2P manual protocol provided by the manufacturer.

#### Whole Exome Sequencing

Whole exome sequencing was performed using the Hiseq property of the NextSeq-500 sequencer (Illumina^®^, San Diego, United States) available in the Bioinformatics Department in the Institute of Brain and Spinal cord (ICM) in Paris, France. High-quality paired-end reads (minimum coverage 100x) were aligned to human reference genome hg19 using the Burrows–Wheeler (BWA-MEM) algorithm v0.7.12 (Li and Durbin, 2009) with default parameters. The resulting BAM files were processed (removal of PCR duplicates, sorting, and indexing) using samtools v1.7 (Li et al., 2009). Variant calling was performed using Freebayes v1.0.0 (Garrison and Marth, 2012) with default parameters, and the resulting VCF files were filtered according to depth (>20) and genotype quality (>20). The VCF file was annotated using SnpEff v4.3t (Cingolani et al., 2012), loaded to Gemini v0.17 (Paila et al., 2013), and then filtered for rare (ExAC MAF <0.01) potentially pathogenic variants that follow an autosomal recessive inheritance pattern.

#### Sanger Sequencing

Sanger sequencing was used to analyze the segregation pattern of candidate variants and confirm the genotypes in affected individuals. Primer3 online tool (Untergasser et al., 2012) was used to design forward and reverse primers. Multiple sequence alignment was performed using Bioedit software v7.2.5 (Hall, 1999) with default parameters.

#### Ethical Consideration

Written informed consent was obtained from each family member (or parents/legal guardians in case of minors) before participation in the study according to the LEUKOFRANCE research program for undetermined leukodystrophies (authorization CPP AU788; CNIL 1406552; AFSSAPS B90298-60).

## Results

**FIGURE 2:**
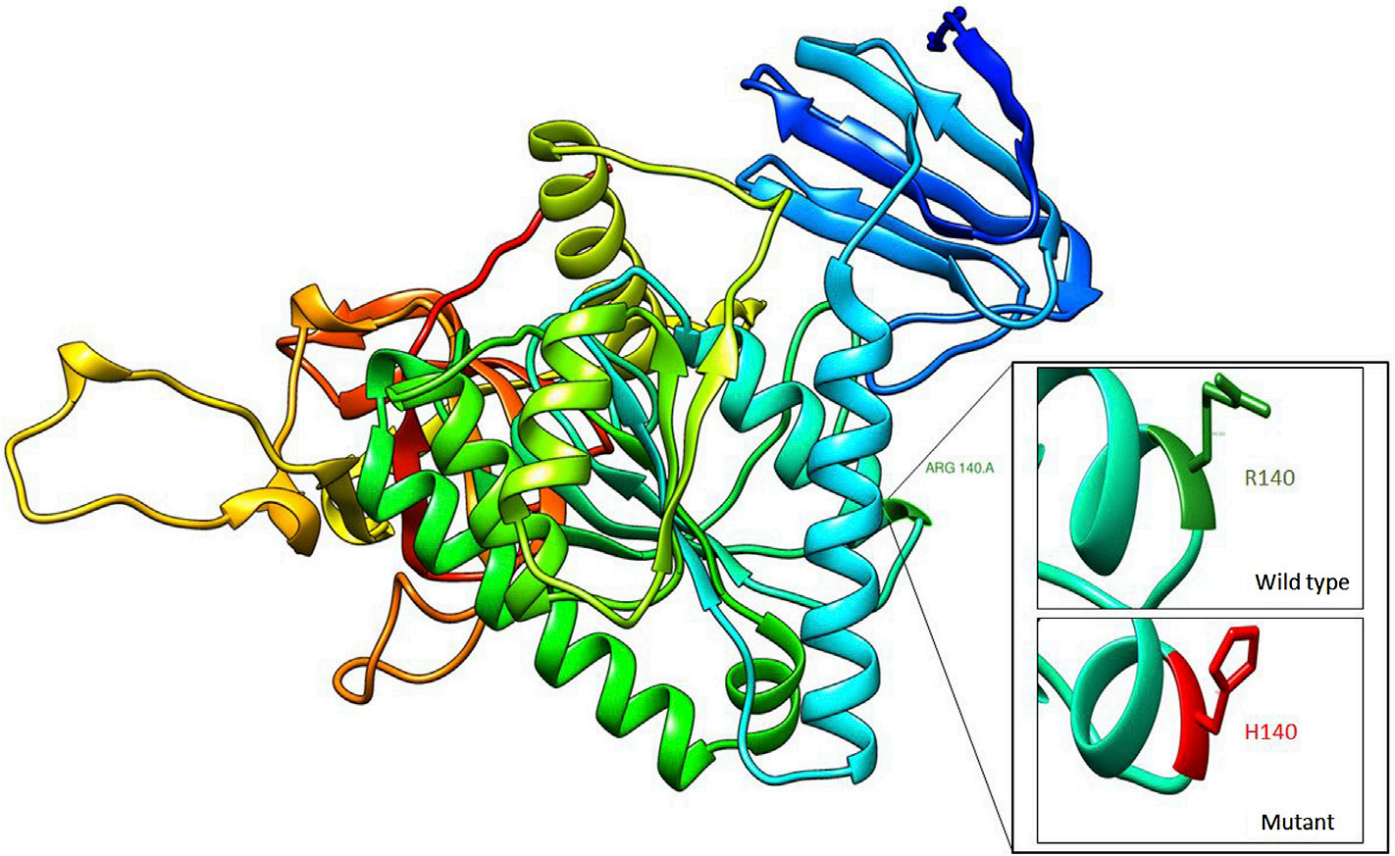
Homology modeling of CLP1 protein showing structural differences between wild type (R140) and mutant residue (H140).

Exome sequencing revealed a homozygous missense pathogenic variant (NM_006831.3:c.419G>A; p.Arg140His) in the *CLP1* gene. The segregation of the variant in the family was consistent with autosomal recessive inheritance (Figure 1). The variant has a very low allele frequency (<0.0001) in the ExAC and gnomAD databases and was predicted to be pathogenic using 11 different pathogenicity prediction tools: SIFT (Ng and Henikoff, 2003), PolyPhen (Adzhubei et al., 2013), MutationTaster (Schwarz et al., 2010), MutationAssessor (Reva et al., 2011), BayesDel_addAF (Feng, 2017), DANN (Quang et al., 2015), EIGEN (Ionita-Laza et al., 2016), FATHMM-MKL (Rogers et al., 2018), LIST-S2 (Malhis et al., 2020), M-CAP (Jagadeesh et al., 2016), and PrimateAI (Sundaram et al., 2018). The wild-type amino acid is highly conserved (Schaffer et al., 2014). Structural differences between the wild type and mutant residues are shown in Figure 2. This pathogenic variant has been reported before in Turkish families with the same clinical presentation (Schaffer et al., 2014) and has been shown to impair CLP1 protein, which is involved in tRNA and mRNA processing leading to the accumulation of unspliced pre-tRNAs (Schaffer et al., 2014). The mutant CLP1 enzyme loses its kinase activity and causes a neurodegenerative phenotype in clp1 null zebrafish (Schaffer et al., 2014), and dysfunction of CLP1 in mice resulted in a similar phenotype of microcephaly, brain atrophy, and neurodegeneration (Karaca et al., 2014).

## Discussion

One of the main distinguishing features of PCH10 is the ethnic origin of the family (Turkish origin) (Rüsch et al., 2020) because PCH10 has not been reported so far from outside Turkey. The disease was first described in 2014 by two independent Turkish studies (Karaca et al., 2014; Schaffer et al., 2014), who reported nine different families with a distinct form of pontocerebellar hypoplasia. Later, another study (Wafik et al., 2018) reported another Turkish family with the same disease. All these families shared the same mutation, a homozygous missense pathogenic variant in the *CLP1* gene, which is involved in the splicing and processing of tRNAs. This study reports a Sudanese family with PCH10 with the same reported variant. All patients with PCH10, including the patients in our study, presented with intellectual disability, microcephaly, and delayed developmental milestones. The patients in our study presented with similar clinical and radiological features reported in the Turkish studies (Table 1). However, the eldest patient in our study presented with a more severe phenotype, including behavioral abnormalities (biting and irritability) and developmental regression. She ultimately succumbed to the disease at the age of 8.5 years following an acute respiratory tract infection. These severe features have not been reported before in patients with PCH10 (Table 1).

All previously reported families originated from Eastern Turkey. Therefore, a founder effect was suggested (Karaca et al., 2014; Schaffer et al., 2014). Schaffer et al. (2014) analyzed the haplotypes of the four Turkish families in their study and estimated from the expected mutation rate and the number of haploblocks in Middle Eastern cohorts that the most recent common ancestor has probably lived around 16 generations ago (+/− 8.7), which coincides with the time of the Ottoman’s expansion. It is possible that the R140H pathogenic variant has been transferred to or from Turkey at this time. However, it is more likely that this mutation arose independently in the Sudanese family, considering the lack of shared genotypes in the region surrounding the mutation (Supplementary Figure S1).

Our study extends the phenotypes of PCH10 and reports for the first time cases with PCH10 of non-Turkish origin.

## Data Availability

The datasets for this article are not publicly available due to concerns regarding participant/patient anonymity. Requests to access the datasets should be directed to the corresponding author.
