# Supplementary material for: Case Report: A New Family With Pontocerebellar Hypoplasia 10 From Sudan
Source: Front Genet. 2022 Jun 2;13:883211. doi: 10.3389/fgene.2022.883211 (PMC9201487; doi:10.3389/fgene.2022.883211)
Supplement: Supplementary file 1 [file DataSheet1.PDF]

Variant	Position	Ref	Alt	Family-1810-6-1	Family-1327-2	Family-1337-2	Family-57718	Sudanese family	Genotype
V1	57236912	G	A	**Het**	NA	NA	HOM	REF	Different genotypes
V2	57309877	G	C	REF	**Het**	NA	HOM	REF	
V3	57313723	G	C	HOM	HOM	HOM	Hom?	REF	Different genotypes
V4	57349911	C	T	HOM	HOM	HOM	HOM	REF	Different genotypes
V5	57368189	A	AG	NA	HOM	HOM	NA	REF	Different genotypes
V6	57369898	G	A	HOM	HOM	HOM	HOM	REF	Different genotypes
V7	57369951	C	T	HOM	HOM	HOM	HOM	REF	Different genotypes
V8	57370065	A	AG	NA	HOM	HOM	NA	HOM	
V9	57379543	A	G	HOM	HOM	HOM	HOM	HOM	shared
V10	57380580	G	T	REF	HOM	HOM	HOM	HOM	
V11	57380702	A	G	HOM	HOM	HOM	HOM	HOM	shared
V12	57387528	G	A	HOM	HOM	HOM	HOM	HOM	
V13	57387815	T	C	HOM	NA	HOM	HOM	HOM	
V14	57388965	A	G	**Het**	HOM	HOM	HOM	REF	Different genotypes
V15	57389932	A	G	HOM	HOM	HOM	HOM	HOM	shared
V16	57416745	C	T	NA	HOM	HOM	NA	HOM	
V17	57476115	A	G	HOM	HOM	NA	HOM	REF	Different genotypes
V18	57500697	T	C	NA	HOM	HOM	HOM	REF	Different genotypes
V19	57542834	A	G	HOM	HOM	HOM	HOM	REF	Different genotypes
V20	57550167	C	T	NA	HOM	HOM	HOM	REF	Different genotypes
V21	57552571	T	C	REF	HOM	HOM	HOM	REF	Different genotypes
V22	57659895	G	A	HOM	HOM	HOM	HOM	HOM	**Disease variant**
V23	57694000	TG	T	NA	Het	NA	HOM	NA	poor coverage
V24	57704669	G	GTTTTTTTT	NA	Het	NA	HOM	NA	poor coverage
V25	57704695	G	T	NA	Het	NA	HOM	NA	poor coverage
V26	57704696	C	T	NA	Het	NA	HOM	NA	poor coverage
V27	57741957	A	G	HOM	HOM	HOM	HOM	HOM	shared
V28	57796519	C	T	HOM	HOM	HOM	HOM	HOM	shared
V29	57803760	C	T	HOM	HOM	HOM	HOM	HOM	shared
V30	58031636	AC	A	NA	HOM	NA	HOM	HOM	shared
V31	58031899	G	C	HOM	HOM	HOM	HOM	REF	Different genotypes
V32	58179792	C	G	HOM	HOM	HOM	HOM	HOM	shared
V33	58191303	C	T	HOM	HOM	HOM	HOM	REF	Different genotypes
V34	58203699	C	T	NA	**Het**	NA	HOM	REF	Different genotypes
V35	58203723	G	A	HOM	HOM	NA	HOM	REF	Different genotypes
V36	58203729	G	A	HOM	HOM	NA	HOM	HOM	
V37	58214757	A	G	HOM	HOM	HOM	HOM	HOM	
V38	58215112	T	C	HOM	HOM	NA	HOM	HOM	
V39	58215148	A	G	HOM	HOM	HOM	HOM	HOM	
V40	58215254	G	T	**Het**	HOM	NA	HOM	HOM	
